# Melatonin for the prevention of postoperative delirium in older adults: a systematic review and meta-analysis

**DOI:** 10.1186/s12877-019-1297-6

**Published:** 2019-10-16

**Authors:** Ashley M. Campbell, David Rhys Axon, Jennifer R. Martin, Marion K. Slack, Lea Mollon, Jeannie K. Lee

**Affiliations:** 1 College of Pharmacy, University of Arizona, 1295 N Martin Avenue, P.O. Box 210202, Tucson, AZ 85721 USA; 2 Arizona Health Sciences Library, Tucson, AZ USA

**Keywords:** Melatonin, Ramelteon, Delirium, Postoperative, Geriatric

## Abstract

**Background:**

Older surgical patients are at high risk of developing postoperative delirium. Non-pharmacological strategies are recommended for delirium prevention, but no pharmacological agents have compelling evidence to decrease the incidence of delirium. The purpose of this study was to assess whether perioperative melatonin decreases the incidence of delirium in older adults undergoing surgical procedures.

**Methods:**

A systematic search using PubMed/Medline, Embase, PsycINFO, CINAHL, and references of identified articles published in English between January 1990 and October 2017 was performed. Two independent reviewers screened titles and abstracts, and then extracted data following a full-text review of included articles with consensus generation and bias assessment. Studies reporting outcomes for melatonin or ramelteon use to prevent delirium in postoperative hospitalized patients (mean age ≥ 50 years) were eligible for inclusion. Data were pooled using a fixed-effects model to generate a forest plot and obtain a summary odds ratio for the outcome of interest (delirium incidence). Cochran’s Q and I^2^ values were used to investigate heterogeneity.

**Results:**

Of 335 records screened, 6 studies were selected for the qualitative analysis and 6 were included in the meta-analysis (*n* = 1155). The mean age of patients in included studies ranged from 59 to 84 years. Patients in intervention groups typically received melatonin or ramelteon at daily doses of two to eight milligrams around cardiothoracic, orthopedic, or hepatic surgeries for one to nine days, starting on the evening before or the day of surgery. The incidence of delirium ranged from 0 to 30% in the intervention groups versus 4–33% in the comparator groups, and was significantly reduced in the melatonin group, with a summary effect of the meta-analysis yielding an odds ratio of 0.63 (95% CI 0.46 to 0.87; 0.006; I^2^ = 72.1%). A one study removed analysis reduced overall odds ratio to 0.310 (95% CI 0.19 to 0.50), while reducing heterogeneity (Cochran’s Q = 0.798, I^2^ = 0.000).

**Conclusion:**

Perioperative melatonin reduced the incidence of delirium in older adults in the included studies. While optimal dosing remains an unanswered question, the potential benefit of melatonin and melatonin receptor agonists may make them a reasonable option to use for delirium prevention in older adults undergoing surgical procedures.

## Background

Delirium is characterized by an acute disturbance in attention with an altered level of consciousness or disorganized thinking that is not better explained by a pre-existing neurocognitive or medical condition, substance intoxication or withdrawal, or medication side effect [1]. Patients who develop delirium in the hospital are at a significantly greater risk of other negative outcomes, including the development of dementia, institutionalization, and one-year mortality [2–4]. One study in post-operative cardiac patients suggested that reduced cognition as a result of post-operative delirium had still not fully recovered, even a year following surgery [5].

While the incidence of delirium in the hospitalized, general medicine population is estimated to be 11–14%, older adults and post-surgical patients are at much greater risk, with an estimated incidence of 20–29% in geriatric medicine patients and 11–51% in surgical patients. Additionally, approximately 56% of hospitalized patients with a history of dementia will develop delirium [6].

Melatonin is a natural hormone produced by the pineal gland in the brain, and is known to be involved in sleep-wake cycle regulation. It has been studied for a variety of uses, including but not limited to insomnia [7–9], jet lag [10–12], circadian rhythm disorders in the blind [13–15], shift work [16–18], and more recently delirium prevention [19, 20]. Recent studies suggest that preoperative cerebrospinal fluid melatonin concentrations may be correlated with delirium risk following hip fracture surgery [21], and that melatonin secretion rapidly declines with age [22].

There is a growing interest in the use of melatonin for the prevention of delirium in hospitalized patients. Hatta and colleagues examined the effect of ramelteon, a selective type 1 and type 2 melatonin receptor agonist, in older adults (aged 65 to 89) on incidence of delirium in hospitalized patients on general medicine and intensive care units. Patients were randomized to receive ramelteon 8 mg (*n* = 34) or placebo (*n* = 33) nightly for 7 days and screened for delirium daily. There was a significantly lower incidence of delirium in the ramelteon group, suggesting that ramelteon may be protective against delirium in hospitalized older patients [19]. Additionally, a 2011 randomized placebo-controlled trial examining the effect melatonin 0.5 mg (*n* = 72) or placebo (*n* = 73) nightly for 14 days or until discharge on incidence of delirium in hospitalized patients aged 65 years or older suggested that patients receiving preventative melatonin experienced a lower risk of delirium (12.0% versus 31.0%, *p* = 0.014) [20]. However, these studies did not include surgical patients.

Given that limited pharmacological interventions are available to prevent post-operative delirium at this time [23, 24], the role of melatonin is worthy of investivation. The purpose of this systematic review and meta-analysis was to assess whether perioperative melatonin administration has an effect on the incidence of delirium in older adults undergoing surgical procedures.

## Methods

### Search strategy

**Fig. 1:**
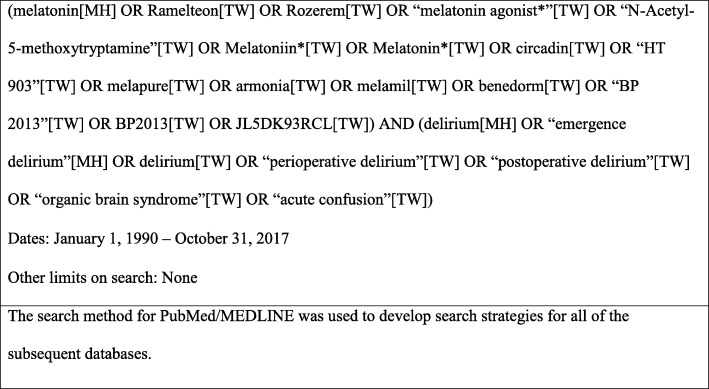
PubMed search strategy

An experienced health sciences librarian developed a comprehensive search strategy with clinical input from the lead authors. An electronic search was systematically conducted in four bibliographic databases: PubMed/Medline (NLM); Embase (Elsevier); PsycINFO (Ebsco); and CINAHL (Ebsco) in November 2017. In addition, the reference lists of obtained articles and relevant systematic reviews were screened, and searches in pertinent websites were conducted to identify further studies that reported melatonin use perioperatively in hospitalized adults from January 1990 to October 2017. This timeframe was selected to coincide with the development of the Beers Criteria, which was initially published in 1991 and categorized potentially inappropriate medications for older adults, including agents that may cause or exacerbate delirium [25]. A combination of controlled vocabulary and keywords were used to search the databases. To identify delirium, terms such as emergence delirium, perioperative delirium, postoperative delirium, organic brain syndrome, and acute confusion were used; while synonymous terms for melatonin such as ramelteon, melatonin receptor agonist, etc. were used to make the search as comprehensive as possible. The search strategy for NLM, which was used to develop search strategies for subsequent databases, is summarized in Fig. 1. The protocol for this study has not been previously published.

### Study selection

**Fig. 2:**
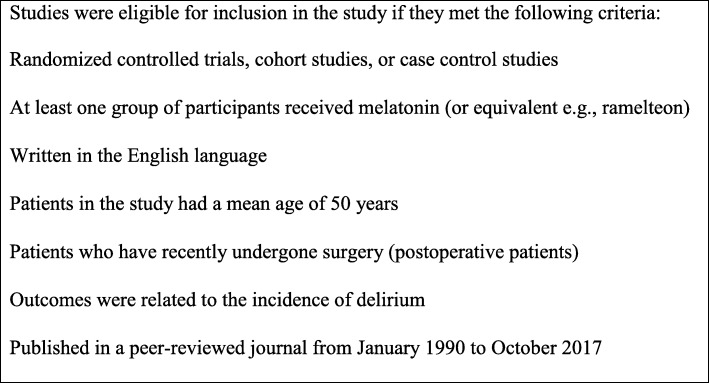
Study inclusion criteria

All identified studies were title screened to identify studies that appeared to address our study aim. Then, two independent reviewers screened each abstract to determine their eligibility for inclusion using an abstract screening tool developed specifically for this study. Abstracts that were conducted before 1990 were excluded from full-text review. Abstracts that were unclear if they met the inclusion criteria were included for full text review to determine eligibility. A data extraction tool was used to identify the final studies for inclusion in the systematic review and meta-analysis. To be included in the systematic review and meta-analysis, studies had to be written in English, report outcomes for melatonin use to prevent delirium in postoperative hospitalized patients with a mean age of 50 or older, and be published in peer-reviewed journals between January 1990 and October 2017. Additionally, the studies also had to be randomized controlled trials, cohort studies, or case control studies with both a melatonin (or equivalent) group and a comparison group. Inclusion and exclusion criteria are summarized in Fig. 2.

### Data extraction

Data were collected on study characteristics, patient characteristics, and study outcomes using a standardized data extraction tool created specifically for this study. Two independent reviewers extracted data from each study report and brought any discrepancies to the research team for resolution through consensus. Study characteristics included: drugs and dose used in the intervention and comparator groups; study duration; study setting; study design; blinding; reasons for surgery; and concurrent diseases and medications. Patient characteristics included: total number included and analyzed; age; and gender. Study outcomes included the incidence of delirium and duration of delirium (if delirium was experienced by the patient).

### Risk of Bias assessment

Risk of bias was assessed using one of two tools depending on the study design. Randomized controlled trials (RCTs) were assessed using the Cochrane risk of bias tool [26]. This tool assessed six bias domains: 1) selection; 2) performance; 3) detection; 4) attrition; 5) reporting; and 6) other bias, which could be reported as having a low, unclear, or high risk of bias [26]. Risk of bias in observational studies was assessed using the Risk of Bias in Non-Randomized Studies – of Interventions (ROBINS-I) assessment tool [27]. This tool assessed seven bias domains: 1) confounding; 2) selection of participants into the study; 3) classification of interventions; 4) deviations from intended interventions; 5) missing data; 6) measurement of outcomes; and 7) selection of the reported result, which could be reported as having a low, moderate, serious, or critical risk of bias [27]. Two investigators independently assessed the risk of bias for each study and scored each domain then met to resolve differences by consensus.

### Outcomes

The primary outcome measure was the incidence of delirium in hospitalized, postoperative patients with a mean age of 50 years or greater.

### Data analysis

Data were entered into comprehensive meta-analysis (CMA; Version 2, Englewood, NJ: Biostat) software for analysis. Given that some outcomes had a value of zero, the CMA software automatically added 0.05 to all values for computation of the odds ratios and variance. With a narrowly defined population and a small number of studies meeting inclusion criteria, many of which had small sample sizes, the possibility exists that the estimate of between studies variance may be over- or underestimated [28]. For these reasons, it did not seem appropriate to extrapolate the findings beyond the included studies, and thus, a fixed effects meta-analysis was used to generate a forest plot and summary odds ratio for the included studies. Cochran’s Q and I^2^ values were used to describe heterogeneity between studies. Stratified analyses were conducted to determine if the incidence of delirium was influenced by study design (observational study versus RCT). Upon examining the findings, a further stratified analysis was performed to investigate the influence of the de Jonghe study (de Jonghe study versus others) [29]. A one study removed analysis was also performed to identify any studies that exerted a greater influence on the outcome. A funnel plot and Kendall’s tau were used to assess publication bias. The a priori alpha level was 0.05.

## Results

### Study selection

**Fig. 3:**
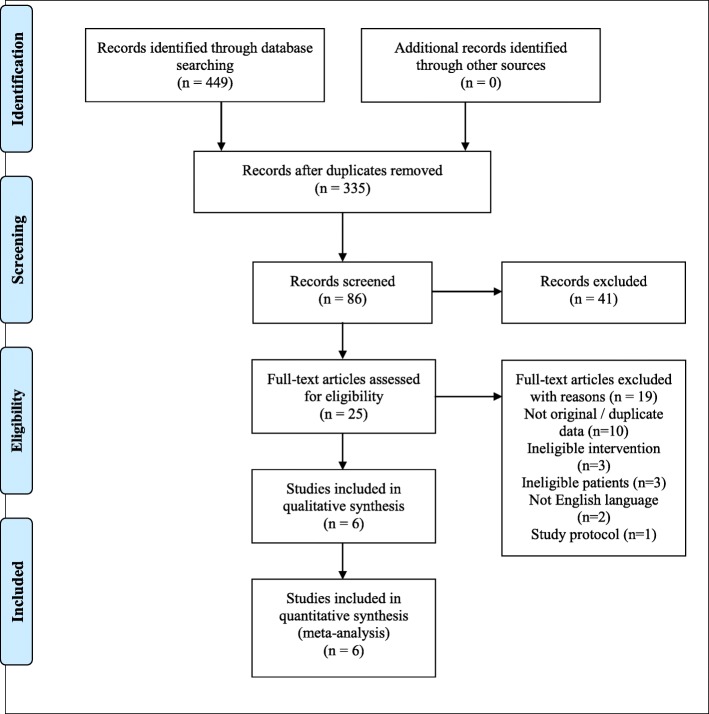
PRISMA 2009 Flow Diagram [35]

The search yielded a total of 335 unique records after the removal of duplicates. Of these, 86 abstracts were screened, 25 articles underwent full-text review, and six were included in the systematic review and meta-analysis [29–34]. The study selection process is described in Fig. 3 [34].

### Characteristics of included studies

Of the six studies included in the study, four were double-blind RCTs [29, 32–34] and two were observational studies [30, 31]. Four studies involved melatonin [29, 30, 32, 33] while two involved ramelteon [31, 34], ,with treatment durations ranging from one to nine days.

In the Miyata study, one patient each in the intervention group and control group had a history of delirium [31], whereas prior delirium was reported in 23.7% of patients in the intervention group and 17.7% of patients in the control group in the de Jonghe study [29]. No patients in the intervention group and one patient in the control group had a history of cognitive impairment in the Miyata study [31], while 55.9% of patients in the intervention group and 55.2% in the control group previously had cognitive impairment in the de Jonghe study [29]. Home benzodiazepine use was reported in the Miyata study (8.3% of individuals in the intervention group versus 22.4% in the control group) and de Jonghe study (9.7% of individuals in the intervention group versus 17.2% in the control group) [29, 31]. In the study by Sultan, all subjects in the intervention (*n* = 53) and control (*n* = 49) groups received spinal anesthesia [33]. Further details of the characteristics of included studies are reported in Table 1, and outcomes are shown in Table 2.

**Table 1:** Characteristics of the studies and study patients

Author (year); Country	Drug (dose)		Duration of Therapy (days)	Study design / (blinding)	Reason for surgery	Patients N		Age Mean (SD) unless otherwise indicated		Male gender N (%)		Scale used to assess delirium	Mean (SD) duration of surgery (minutes)	
	I	C				I	C	I	C	I	C		I	C
Artemiou (2015); Slovakia [30]	Melatonin (5 mg)	No treatment	5	Prospective observational (none)	Cardiac	250	250	64.3 (±10.1)	65.2 (±10.3)	179 (71.6)	171 (68.4)	CAM-ICU	NR	NR
de Jonghe (2014); The Netherlands [29]	Melatonin (3 mg)	Placebo	5	RCT (double)	Hip fracture	186	192	Mean (SD) 84.1 (±8)	Mean (SD) 83.4 (±7.5)	53 (28.5)	62 (32.3)	DSM-IV	NR	NR
Miyata (2017); Japan [31]	Ramelteon (8 mg)	Placebo	7	Retrospective chart review (none)	Pulmonary resection	24	58	79 (70–89)^a^	Median (Range) 76.5 (70–87)	21 (88)	43 (74)	ICDSC	293 (±108)	280 (±91.4)
Nickkholgh (2011); Germany [32]	Melatonin (50 mg/kg)	Placebo	1	RCT (double)	Liver resection	25	23	Mean (SD) 59 (±10)	Mean (SD) 56 (±11)	17 (68)	11 (48)	NR	202 (±80)	212 (±79)
Sultan (2010); Egypt [33]	Melatonin (5 mg)	No treatment	2	RCT (double)	Hip arthroplasty	53	49	Mean (SD) 70.4 (±7.1)	Mean (SD) 72.3 (±6.4)	24 (45.3)	22 (44.9)	AMT	126.8 (±44.9)	119.7 (±36.7)
Yamaguchi (2014); Japan [33]^b^	Ramelteon (8 mg)	Placebo	4	RCT (double)	Total knee arthroplasty	22	23	≥70	≥70	NR	NR	ICDSC	NR	NR

Abbreviations

*I* intervention group

*C* comparison group

*Mg* milligrams

*Kg* kilograms

*NA* Not applicable

*RCT* randomized controlled trial

*SD* standard deviation

*NR* not reported

*CAM-ICU* Confusion Assessment Method for the Intensive Care Unit

*DSM-IV* delirium observation screening scale

*ICDSC* intensive care delirium screening checklist score (delirium >/= 4)

*AMT* abbreviated mental test (delirium < 8)

Footnotes:

^a^Median (range).

^b^Abstract only.

**Table 2:** Study outcomes

Author (year); Country	Delirium incidence N (%)		Delirium duration (days) median (range)		Hospital stay (days) Mean (SD) unless otherwise indicated	
	I	C	I	C	I	C
Artemiou (2015); Slovakia [30]	21 (8.4)	52 (20.8)	NR	NR	9.54 (±3.89)	10.84 (±6.85)
de Jonghe (2014); The Netherlands [29]	55 (29.6)	49 (25.5)	2 (1–3)	2 (1–3)	11 (6–14.5)^a^	11 (8–17)^a^
Miyata (2017); Japan [31]	0	5 (8.6)	NR	NR	13.5 (±9.62)	13.6 (±7.81)
Nickkholgh (2011); Germany [32]	0	1 (4.3)	NR	NR	13.5 (±1.5)	17 (±2)
Sultan (2010); Egypt [33]	5 (9.4)	16 (32.7)	NR	NR	NR	NR
Yamaguchi (2014); Japan [34]	0	2 (8.7)	NR	NR	NR	NR

Footnotes:

^a^ Median (Range)

Abbreviations

*I* intervention group

*C* comparison group

*NR* not reported

*NA* Not applicable

*SD* standard deviation

### Results of individual studies

**Fig. 4:**
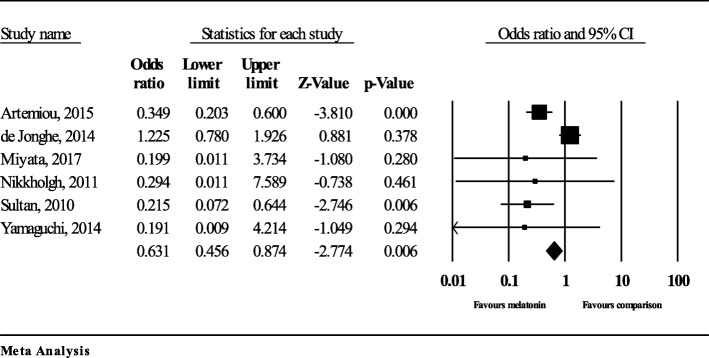
Forest plot of melatonin studies for the prevention of delirium in postoperative patients

Figure 4 presents the odds ratios for each study included in the meta-analysis. Odds ratios ranged from 0.19 (95% confidence interval [CI] = 0.01 to 4.21) to 1.23 (95% CI = 0.78 to 1.93) and indicate the odds of a patient experiencing delirium if they took melatonin as a preventive measure compared to placebo/control. For example, the de Jonghe article indicated that patients taking melatonin had an odds ratio of 1.2 (i.e., greater odds) of experiencing delirium compared to those taking placebo, although the remaining studies indicated that patients taking melatonin had lower odds of experiencing delirium compared to placebo [29]. Statistically significant differences were detected between the intervention and control/placebo groups in the studies by Artemiou (*p* < 0.001) and Sultan (*p* = 0.006) [30, 33], but not in the remaining studies (*p* > 0.05) [29, 31, 32, 34].

### Synthesis of results

**Fig. 5:**
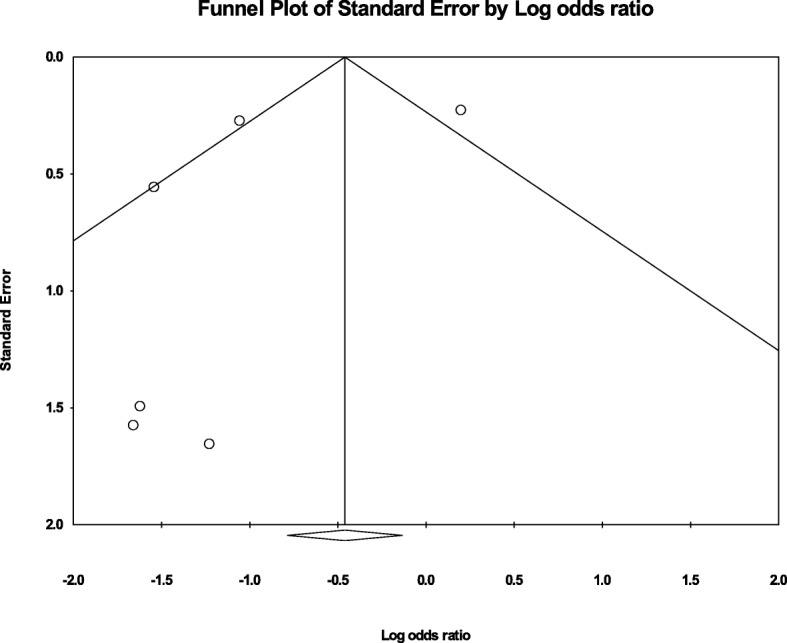
Meta-analysis funnel plot

The summary effect of the six included studies shown in Fig. 4 gives an odds ratio of 0.63 (95% CI = 0.46 to 0.87), indicating that patients taking melatonin had lower odds of experiencing delirium compared to patients taking placebo. There was evidence of publication bias based on visual inspection of the Funnel Plot (Fig. 5); however, Kendall’s tau was not significant (*p* = 0.775).

There was evidence of heterogeneity among the data, with a Cochran’s Q value of 17.95 (five degrees of freedom; *p* = 0.003) and an I^2^ value of 72.14. Visual inspection of the forest plot indicated differences between the de Jonghe study and other studies, so a stratified analysis of the de Jonghe study versus the remaining studies was conducted. The difference in effect for the de Jonghe study versus other studies was significant (*p* < 0.001). A stratified analysis was also conducted by study design (RCTs versus observational studies), but this was not found to have a significant impact on the incidence of delirium (*p* = 0.69).

In the one study removed analysis, exclusion of the de Jonghe study reduced the overall odds ratio to 0.310 (95% CI 0.19 to 0.50), and also reduced the amount of heterogeneity present (Cochran’s Q = 0.798, I^2^ = 0.000).

### Risk of Bias in included studies

The risk of bias assessment is summarized in Table 3. Of note among the RCTs, attrition bias was considered high in the de Jonghe study, and detection bias was considered high in the Nickkholgh study. Since only an abstract was available for the Yamaguchi article, it was difficult to fully assess the risk of bias; thus, most risk of bias domains were graded as unclear. In both observational studies, bias due to confounding was considered a moderate risk overall [30, 31], although the Artemiou study used multiple logistic regression to identify independent risk factors for delirium whereas the Miyata study did not. In all other instances the risk of bias was considered low or moderate.

**Table 3:** Risk of bias assessment for included studies

Author (year); Country							
*Randomized controlled trials*	Selection bias	Performance bias	Detection bias	Attrition bias	Reporting bias	Other bias	
de Jonghe (2014); The Netherlands [29]	Low	Low	Low	High	Low	Low	
Nickkholgh (2011); Germany [32]	Low	Low	High	Low	Unclear	Unclear	
Sultan (2010); Egypt [33]	Unclear	Low	Unclear	Unclear	Low	Low	
Yamaguchi (2014); Japan [34]	Unclear	Unclear	Unclear	Unclear	Low	Unclear	
*Observational studies*	Bias due to confounding	Bias in selection of participants into the study	Bias in classification of interventions	Bias due to deviations from intended interventions	Bias due to missing data	Bias in measurement of outcomes	Bias in selection of the reported results
Artemiou (2015); Slovakia [30]	Moderate	Low	Low	Low	Low	Moderate	Low
Miyata (2017); Japan [31]	Moderate	No information	Low	Low	Low	Low	Low

## Discussion

Currently, there is a very small body of literature examining the use of melatonin for the prevention of postoperative delirium in older adults. In conjunction with clinician input, the exhaustive literature search was driven by a health sciences librarian who is highly experienced in systematic reviews. While the body of literature is small at this time, the findings of this meta-analysis suggest that perioperative melatonin administration may significantly reduce the incidence of delirium in older adults undergoing surgical procedures – a common, yet detrimental complication of surgery in older adults. The odds ratio was highly sensitive to removal of the one study that did not have results in favor of melatonin reducing risk of delirium, resulting in a reduction of the odds ratio from 0.63 to 0.31, and heterogeneity to zero [29]. One possible explanation for the negative results in this particular study may be due discrepancy among the two study groups related to prior delirium. Even though statistics were not reported for the baseline characteristics, the group receiving melatonin appeared to have a greater percentage of patients with a history of delirium (23.7% versus 17.2% in the placebo group), which is a well-established risk factor for developing delirium [6, 36]. Additionally, the authors of the de Jonghe study noted that there was an increased probability of type 2 error, as they had a large number of patients who were excluded post-randomization (withdrawal of consent, delirium at admission, loss to follow-up, etc.). Only 378 patient data were analyzed, while 444 patients were randomized, increasing risk of attrition bias [29].

While the Kendall’s tau coefficient (*p* = 0.78) is non-significant, visual inspection of the funnel plot appeared to indicate publication bias in the present meta-analysis. This is likely due to the small number of studies that were eligible for inclusion in the meta-analysis.

There are currently three sets of clinical guidelines that can be applied to the management of delirium in the inpatient setting: The American Geriatrics Society (AGS) Clinical Practical Guideline for Postoperative Delirium in Older Adults; the Society of Critical Care Medicine Clinical Practice Guideline on Management of Pain, Agitation, and Delirium (PAD) in Adult Patients in Intensive Care Unit; and the American Psychiatric Association (APA) Practice Guideline [37–39]. None of the three guidelines made recommendations for pharmacological intervention for the prevention of delirium. The AGS guidelines specifically acknowledge melatonin as being an agent considered for the prevention of delirium, but noted that their practice guidelines did not address it due to lack of evidence [37]. The PAD guidelines also state that there is a lack of data for an agent that effectively prevents delirium [38].

This meta-analysis has several limitations. First, the few studies that met inclusion criteria were small, and not all met their predefined statistical power [29]. By using a fixed effects model for this narrowly defined population, the findings presented are restricted to the population included in the identified studies and are not generalizable at this time. In addition, many well-known predisposing and precipitating risk factors for delirium were not reported in all studies [40]. For example, time from admission to surgery, body mass index, cognitive impairment, depression, uncontrolled pain, intraoperative blood loss, baseline functional status, and use of medications that could increase delirium risk [6, 40–46] are all possible risk factors for developing delirium. Thus, it is difficult to assess whether intervention and control groups were at similar baseline risk.

Another limitation is the heterogeneity of included studies with regards to type of surgical procedure and melatonin dosing. Because melatonin is not regulated by the United States Food and Drug Administration, dose standardization is not guaranteed unless the manufacturers voluntarily submit their products for strength and purity testing [47]. Assuming that the doses reported were accurately reflecting the contents of the studied products, one potential explanation for the negative result in the de Jonghe study is that the melatonin dose was lower than the other (3 mg versus 5 mg or more) [29]. Additionally, while the requirement for an average age of 50 years and older increased the number of studies meeting inclusion criteria, it also led to a wide age variability. Given the small number of studies, it is difficult to ascertain whether older age (e.g.: 85 years and older versus 65 and older) had any effect on benefit, though the study with the oldest average age (84.1 and 83.4 in the intervention and control groups, respectively) was the one study that did not show benefit with melatonin [29]. While this study suggests that melatonin may have a protective effect against delirium when used in the individuals included in these studies, optimal dosing and ages at which patients are conferred the greatest benefit have yet to be determined.

One of the most prominent sources of variability among included studies was in delirium assessment methods. The Confusion Assessment Method is the current gold standard for delirium assessment, but many studies used alternative tools [48]. One study included delirium as a secondary outcome, and it is unclear from the methodology report whether this was a pre-determined endpoint and how regularly potential incidence of delirium was assessed.

Melatonin remains an agent of interest due to is its relatively benign side effect profile. A 2005 systematic review and meta-analysis of ten studies involving 222 patients receiving melatonin for the treatment of primary sleep disorders reported 13 headaches, 10 cases of dizziness, 3 cases of nausea, and 3 cases of drowsiness in the cohort of subjects receiving melatonin [49]. When compared to placebo, the incidence of these events was not statistically significant.

With relatively few side effects and minimal pharmacologic options for the prevention of delirium, melatonin continues to be an agent of interest. While preparing this manuscript, our literature search yielded a 2002 case report of melatonin 2 mg daily used to successfully prevent post-operative delirium in a male patient with a history of delirium undergoing surgery on an infected knee joint [50]. We also identified one published protocol aiming to assess the efficacy of melatonin 3 mg versus placebo daily for the prevention of postoperative delirium in patients aged 50 and older who are undergoing cardiac surgery [51]. Another ongoing study aims to examine the potential benefits of melatonin related to delirium in high-risk palliative oncology inpatients; however, this study is not specific to the surgical population [52]. Future research should re-examine this question when additional, more comprehensive data are available to obtain a more reliable estimate.

## Conclusions

Currently, where there is no supported pharmacological intervention to prevent delirium incidence in older surgical patients, this meta-analysis suggests that melatonin may become that first agent. The odds of developing delirium in patients who received melatonin agonists perioperatively were 37% less (*p* = 0.006) than those who received placebo or no treatment at all. Although optimal dosing remains an unanswered question, the potential benefit, low cost, and benign side effect profile, make melatonin an attractive option to use in older adults undergoing surgical procedures to reduce delirium incidence.

## Data Availability

No new data were created during this study.

## Abbreviations

AGS: American Geriatrics Society
APA: American Psychiatric Association
CI: Confidence interval
PAD: Pain, Agitation, and Delirium
RCT: Randomized controlled trials
ROBINS-I: Risk of Bias in Non-Randomized Studies – of Interventions

## References

[1] American Psychiatric Association, Diagnostic and Statistical Manual, 5th ed, APA Press, Washington, DC; 2013.

[2] Pitkala KH, Laurila JV, Strandberg TE, Tilvis RS (2005). Prognostic significance of delirium in frail older people. Dement Geriatr Cogn Disord.

[3] Buurman BM, Hoogerduijn JG, de Haan RJ (2011). Geriatric conditions in acutely hospitalized older patients: prevalence and one-year survival and functional decline. PLoS One.

[4] Leslie DL, Zhang Y, Holford TR, Bogardus ST, Leo-Summers LS, Inouye SK (2005). Premature death associated with delirium at 1-year follow-up. Arch Intern Med.

[5] Saczynski JS, Marcantonio ER, Quach L (2012). Cognitive trajectories after postoperative delirium. N Engl J Med.

[6] Inouye SK, Westendorp RG, Saczynski JS (2014). Delirium in elderly people. Lancet.

[7] Ferracioli-Oda E, Qawasmi A, Bloch MH (2013). Meta-analysis: melatonin for the treatment of primary sleep disorders. PLoS One.

[8] Garfinkel D, Laudon M, Nof D, Zisapel N (1995). Improvement of sleep quality in elderly people by controlled-release melatonin. Lancet.

[9] Buscemi N, Vandermeer B, Hooton N (2005). The efficacy and safety of exogenous melatonin for primary sleep disorders. A meta-analysis. J Gen Intern Med.

[10] Petrie K, Dawson AG, Thompson L, Brook R (1993). A double-blind trial of melatonin as a treatment for jet lag in international cabin crew. Biol Psychiatry.

[11] Petrie K, Conaglen JV, Thompson L, Chamberlain K (1989). Effect of melatonin on jet lag after long haul flights. BMJ.

[12] Herxheimer A, Petrie KJ (2002). Melatonin for the prevention and treatment of jet lag. Cochrane Database Syst Rev.

[13] Sack RL, Brandes RW, Kendall AR (2000). Entrainment of free-running circadian rhythms by melatonin in blind people. N Engl J Med.

[14] Palm L, Blennow G, Wetterberg L (1997). Long-term melatonin treatment in blind children and young adults with circadian sleep-wake disturbances. Dev Med Child Neurol.

[15] Hack LM, Lockley SW, Arendt J, Skene DJ (2003). The effects of low-dose 0.5-mg melatonin on the free-running circadian rhythms of blind subjects. J Biol Rhythm.

[16] Wright SW, Lawrence LM, Wrenn KD (1998). Randomized clinical trial of melatonin after night-shift work: efficacy and neuropsychologic effects. Ann Emerg Med.

[17] Buscemi N, Vandermeer B, Hooton N (2006). Efficacy and safety of exogenous melatonin for secondary sleep disorders and sleep disorders accompanying sleep restriction: meta-analysis. BMJ.

[18] Jorgensen KM, Witting MD (1998). Does exogenous melatonin improve day sleep or night alertness in emergency physicians working night shifts?. Ann Emerg Med.

[19] Hatta K, Kishi Y, Wada K (2014). Preventive effects of ramelteon on delirium: a randomized placebo-controlled trial. JAMA Psychiatry.

[20] Al-aama T, Brymer C, Gutmanis I, Woolmore-goodwin SM, Esbaugh J, Dasgupta M (2011). Melatonin decreases delirium in elderly patients: a randomized, placebo-controlled trial. Int J Geriatr Psychiatry.

[21] Scholtens RM, de Rooij SE, Vellekoop AE, Vrouenraets BC, van Munster BC (2016). Preoperative CSF Melatonin Concentrations and the Occurrence of Delirium in Older Hip Fracture Patients: A Preliminary Study. PLoS ONE.

[22] Zhdanova, IV, Wurtman, RJ. In: Endocrinology: Basic and Clinical Principles, PM, Conn, S, Melmed (Eds), Humana Press, Inc, Totowa, NJ, 1997. p. 281. Copyright © 1997 Humana Press.

[23] Oh ES, Fong TG, Hshieh TT, Inouye SK (2017). Delirium in older persons: advances in diagnosis and treatment. JAMA.

[24] Neufeld KJ, Yue J, Robinson TN, Inouye SK, Needham DM (2016). Antipsychotic medication for prevention and treatment of delirium in hospitalized adults: a systematic review and meta-analysis. J Am Geriatr Soc.

[25] Beers MH, Ouslander JG, Rollingher I, Reuben DB, Brooks J, Beck JC (1991). Explicit criteria for determining inappropriate medication use in nursing home residents. UCLA division of geriatric medicine. Arch Intern Med.

[26] Higgins JP, Altman DG, Gotzsche PC, Juni P, Moher D, Oxman AD, Savovic J, Schulz KF, Weeks L, Sterne JA, Cochrane Bias methods G (2011). Cochrane Statistical Methods G The Cochrane Collaboration's tool for assessing risk of bias in randomised trials. BMJ.

[27] Sterne JA, Hernan MA, Reeves BC (2016). ROBINS-I: a tool for assessing risk of bas in non-randomised studies of interventions. BMJ.

[28] Borenstein M, Hedges LV, Higgins JP, Rothstein HR (2009). Introduction to meta-analysis.

[29] de Jonghe A, van Munster BC, Goslings JC (2014). Effect of melatonin on incidence of delirium among patients with hip fracture: a multicentre, double-blind randomized controlled trial. CMAJ.

[30] Artemiou P, Bily B, Bilecova-Rabajdova M (2015). Melatonin treatment in the prevention of postoperative delirium in cardiac surgery patients. Kardiochir Torakochirurgia Pol.

[31] Miyata R, Omasa M, Fujimoto R, Ishikawa H, Aoki M (2017). Efficacy of Ramelteon for delirium after lung cancer surgery. Interact Cardiovasc Thorac Surg.

[32] Nickkholgh A, Schneider H, Sobirey M (2011). The use of high-dose melatonin in liver resection is safe: first clinical experience. J Pineal Res.

[33] Sultan SS (2010). Assessment of role of perioperative melatonin in prevention and treatment of postoperative delirium after hip arthroplasty under spinal anesthesia in the elderly. Saudi J Anaesth.

[34] Yamaguchi Y, Mihara T, Taguri M, Yamaguchi O, Goto T (2014). Melatonin receptor agonist for the prevention of postoperative delirium in elderly patients: a randomized, double-blind, placebo-controlled trial. Intensive Care Med.

[35] Moher D, Liberati A, Tetzlaff J, Altman DG, The PRISMA Group (2009). Preferred reporting items for systematic reviews and meta-analyses: the PRISMA statement. PLoS Med.

[36] Litaker D, Locala J, Franco K, Bronson DL, Tannous Z (2001). Preoperative risk factors for postoperative delirium. Gen Hosp Psychiatry.

[37] American Geriatrics Society Expert Panel on Postoperative Delirium in Older Adults. American Geriatrics Society abstracted clinical practice guideline for postoperative delirium in older adults. J Am Geriatr Soc. 2015;63(1):142-50. 10.1111/jgs.13281. 10.1111/jgs.13281 PMC5901697 25495432

[38] Barr J, Fraser GL, Puntillo K (2013). Clinical practice guidelines for the management of pain, agitation, and delirium in adult patients in the intensive care unit. Crit Care Med.

[39] American Psychiatric Association: Practice guideline for the treatment of patients with delirium. Am J Psychiatry. 1999;156(5 Suppl):1-20. https://link.springer.com/content/pdf/10.1186%2F1471-2318-14-96.pdf. 10327941

[40] Inouye SK, Charpentier PA (1996). Precipitating factors for delirium in hospitalized elderly persons: predictive model and interrelationship with baseline vulnerability. JAMA.

[41] Inouye SK, Viscoli CM, Horwitz RI, Hurst LD, Tinetti ME (1993). A predictive model for delirium in hospitalized elderly medical patients based on admission characteristics. Ann Intern Med.

[42] Marcantonio ER, Goldman L, Mangione CM (1994). A clinical prediction rule for delirium after elective noncardiac surgery. JAMA.

[43] Juliebø V, Bjøro K, Krogseth M, Skovlund E, Ranhoff AH, Wyller TB (2009). Risk factors for preoperative and postoperative delirium in elderly patients with hip fracture. J Am Geriatr Soc.

[44] Lynch EP, Lazor MA, Gellis JE, Orav J, Goldman L, Marcantonio ER (1998). The impact of postoperative pain on the development of postoperative delirium. Anesth Analg.

[45] Marcantonio ER, Juarez G, Goldman L (1994). The relationship of postoperative delirium with psychoactive medications. JAMA.

[46] Marcantonio ER, Goldman L, Orav EJ, Cook EF, Lee TH (1998). The association of intraoperative factors with the development of postoperative delirium. Am J Med.

[47] The United States Pharmacopeia. Dietary Supplements Verification Program. http://www.usp.org/verification-services/dietary-supplements-verification-program.

[48] Inouye SK, van Dyck CH, Alessi CA, Balkin S, Siegal AP, Horwitz RI. Clarifying confusion: the confusion assessment method. A new method for detection of delirium. Ann Intern Med 1990;113(12):941–948. 10.7326/0003-4819-113-12-941 2240918

[49] Buscemi N, Vandermeer B, Hooton N (2005). The efficacy and safety of exogenous melatonin for primary sleep disorders. A meta-analysis. J Gen Intern Med.

[50] Hanania M, Kitain E (2002). Melatonin for treatment and prevention of postoperative delirium. Anesth Analg.

[51] Ford AH, Flicker L, Passage J (2016). The healthy heart-mind trial: melatonin for prevention of delirium following cardiac surgery: study protocol for a randomized controlled trial. Trials.

[52] Bush SH, Lacaze-masmonteil N, Mcnamara-kilian MT (2016). The preventative role of exogenous melatonin administration to patients with advanced cancer who are at risk of delirium: study protocol for a randomized controlled trial. Trials.

